# Multiplicity adjustments in parallel-group multi-arm trials sharing a control group: Clear guidance is needed

**DOI:** 10.1016/j.cct.2021.106656

**Published:** 2022-02

**Authors:** Síle F. Molloy, Ian R. White, Andrew J. Nunn, Richard Hayes, Duolao Wang, Thomas S. Harrison

**Affiliations:** a Institute for Infection and Immunity, St George's University of London, London, UK; b MRC Clinical Trials Unit at UCL, Institute of Clinical Trials and Methodology, University College London, London, UK; c Department of Infectious Disease Epidemiology, London School of Hygiene and Tropical Medicine, London, UK; d Global Health Trials Unit, Department of Clinical Sciences, Liverpool School of Tropical Medicine, Liverpool, UK

**Keywords:** Multi-arm, parallel-group clinical trials, Multiplicity, Type 1 error, Family-wise error rate (FWER)

## Abstract

Multi-arm, parallel-group clinical trials are an efficient way of testing several new treatments, treatment regimens or doses. However, guidance on the requirement for statistical adjustment to control for multiple comparisons (type I error) using a shared control group is unclear. We argue, based on current evidence, that adjustment is not always necessary in such situations. We propose that adjustment should not be a requirement in multi-arm, parallel-group trials testing distinct treatments and sharing a control group, and we call for clearer guidance from stakeholders, such as regulators and scientific journals, on the appropriate settings for adjustment of multiplicity.

Multiplicity is a major consideration in the analysis of clinical trials. It occurs when multiple significance tests are carried out, increasing the family-wise error rate (FWER), the probability of a “false positive” statistically significant result or type 1 error. Multiplicity can arise for various reasons including use of multiple outcomes, repeated measures, interim analyses, multiple sub-groups, factorial designs and, the focus of this viewpoint, multi-arm clinical trials. It is widely accepted that control of multiplicity is essential in many situations, for example, early stopping of a trial based on interim analyses, or where more than one hypothesis is being tested within a family of hypotheses [1,2]. If not handled correctly, unsubstantiated claims for the effectiveness of a drug may be made due to an inflated rate of false positive conclusions, and this is especially important in confirmatory trials for licensed medications [1,3]. Various methods of control have been developed including hierarchical procedures, the Bonferroni Method and Dunnett's test [4]. However, if such adjustments are applied unnecessarily, potentially effective treatments may be discarded prematurely [5].

Multi-arm trial designs are valuable in clinical research. They allow a number of new treatments, or varying treatment regimens/doses, to be tested within a single trial, increasing efficiency and reducing costs associated with conducting several independent trials. A three-arm trial, for instance, reduces the sample size that would be required for two independent trials by 25% due to efficient sharing of the control group. Some 20% of superiority trials registered in 2010–2012 had more than two groups [6]. However, there appears no consensus, across stakeholders such as regulators and scientific journals, on the necessity to control for a potentially inflated type 1 error rate when comparing distinct treatments to a shared control group in confirmatory parallel-group multi-arm trials [7] and this has become the subject of much recent debate among statisticians and trialists [5,[8], [9], [10], [11]]. Many guidelines on multiplicity do not refer specifically to multi-arm trials [[1], [2], [3]], resulting in inconsistencies in the application of adjustment methods in published articles. A 2014 review found that 49% of published multi-arm trials reported using a multiple-testing adjustment, with adjustment more common in trials evaluating multiple doses or regimens of the same treatments (67%), but surprisingly there was little evidence of difference in adjustment between exploratory and confirmatory trials [10].

There is a general consensus that for multi-arm exploratory trials stringent multiple-testing adjustment is not required [10] as doing so may drop potentially effective treatments too early in the assessment process. Conversely, many authors agree with current guidance from the FDA and EMA that for confirmatory trials where arms represent several doses or regimens of the same treatment, adjustments for multiplicity should be applied [5,[12], [13], [14], [15]]. However, the literature is unclear on the necessity of adjustment in confirmatory trials where the different arms represent distinct treatments which are compared with a shared control [10]. A number of authors argue that adjustment is not always necessary, particularly where the results are not combined into one final conclusion and decision [10,[16], [17], [18]], with Parker et al. arguing that non-adjustment should be the default starting point in such situations [15]. By contrast, guidance from the New England Journal of Medicine requires adjustment in this scenario, even for exploratory analyses, though no rationale is given for applying the same rules to all types of multiplicity [2].

In many cases where adjustment for multiple testing is required, the tests are correlated: for example, when tests of multiple outcomes in the same trial are correlated. Correlation also arises between the multiple tests in a multi-arm trial when they share a common control group. This correlation has practical implications: it makes Dunnett's test the best way to control for multiplicity, it reduces the impact of multiplicity on the FWER [5,19,20], and it increases the probability of making multiple errors given that at least one error is made [11]. However, the correlation does not inform whether multiplicity should be controlled [5].

The key issue in determining whether to control for multiplicity is whether multiple tests are conceptually related: that is, how separate are the scientific questions or the claims to be made? If an issue of a journal publishes multiple clinical trials relating to different medical areas, then the overall type 1 error rate is increased, but no-one would suggest controlling for multiplicity. If multiple doses of the same drug are tested, on the other hand, a claim of efficacy of the drug could be made if any one dose shows benefit, so multiplicity should be controlled: this is true whether the doses are tested in the same trial or in separate trials. Similarly, if multiple treatments are investigated for the purpose of a single regulatory submission then this may be a reason to control the FWER [21]. If drugs with different mechanisms of action are evaluated in the same trial, we believe that control for multiplicity is not required, just as if they were evaluated in separate trials. Hence the “family” over which FWER should be controlled is usually a treatment, and the difficult question is whether closely related treatments should be included in the same family: for example, drugs of the same class, or similar multi-drug regimens [21].

An alternative way to account for multiple tests is to control the false discovery rate (FDR), the expected proportion of rejected null hypotheses that are actually true. The FDR is similar to the FWER in studies with small numbers of experimental treatments (e.g. up to three), but is less stringent than the FWER with larger numbers of experimental treatments (e.g. five or more) [22]. Controlling the FDR may be done using Benjamini–Hochberg procedures [22,23]. When multiple drugs are successful, the FDR has the advantage that it represents the expected proportion of inefficacious drugs among the successful drugs. Wason et al. recommend that sponsors and trialists consider use of the FDR for multi-arm trials testing distinct treatment arms [22], while others suggest the FDR as an appropriate control measure in the context of trials with a large number of treatment arms [24]. However, trials with many treatments may be less likely to have distinct treatments.

We have focussed on the parallel-group multi-arm trial design, but various perspectives on the need for multiplicity adjustments in more complex designs such as basket, umbrella and platform trials are also being debated [14,[24], [25], [26], [27], [28]]. Such new and adaptive designs are increasingly important, especially in the case of COVID-19 where adaptive, cost-effective and rapid trials are critical [24]. However, as discussed by Collignon et al. [25], these complex designs have raised challenging statistical questions around the need for control of multiple testing when adding arms or drugs over time.

In conclusion, increasing trial complexity makes addressing multiplicity more complex but also more important. Clearer guidance for trialists from stakeholders, such as regulators and scientific journals, on the appropriate settings for adjustment of multiplicity is required. We agree with others that the need to adjust or not should be well justified based on the complexity of design and the specific setting and objectives of each trial [15,21] and that control of the FDR should be considered for trials testing a large number of treatments [22]. We propose that, for simple parallel-group multi-arm trials of distinct treatments with a shared control, adjustment should not be a requirement. However, further clarity is needed to define what are distinct treatments [21,25].

## Declaration of Competing Interest

The authors declare that they have no known competing financial interests or personal relationships that could have appeared to influence the work reported in this paper.
